# Stromal derived factor-1 exerts differential regulation on distinct cortical cell populations *in vitro*

**DOI:** 10.1186/1471-213X-7-31

**Published:** 2007-04-10

**Authors:** James Pritchett, Clare Wright, Leo Zeef, Bagirathy Nadarajah

**Affiliations:** 1 1.124 Stopford Building, Faculty of Life Sciences, The University of Manchester, Manchester M13 9PT, UK

## Abstract

**Background:**

Stromal derived factor (SDF-1), an alpha chemokine, is a widely known chemoattractant in the immune system. A growing body of evidence now suggests multiple regulatory roles for SDF-1 in the developing nervous system.

**Results:**

To investigate the role of SDF-1 signaling in the growth and differentiation of cortical cells, we performed numerous in *vitro *experiments, including gene chip and quantitative RT-PCR analysis. Using SDF-1 medium and AMD3100, a receptor antagonist, we demonstrate that the chemokine signaling regulates key events during early cortical development. First, SDF-1 signaling maintains cortical progenitors in proliferation, possibly through a mechanism involving connexin 43 mediated intercellular coupling. Second, SDF-1 signaling upregulates the differentiation of cortical GABAergic neurons, independent of sonic signaling pathway. Third, SDF-1 enables the elongation and branching of axons of cortical glutamatergic neurons. Finally, cortical cultures derived from CXCR4-/- mutants show a close parallel to AMD3100 treatment with reduced cell proliferation and differentiation of GABAergic neurons.

**Conclusion:**

Results from this study show that SDF-1 regulates distinct cortical cell populations *in vitro*.

## Background

The cerebral cortex is primarily composed of glutamatergic pyramidal neurons of dorsal telencephalon and GABAergic interneurons that emanate from ventral telencephalon [1]. The generation of cortical neurons is a tightly orchestrated process that involves the commitment of multipotent stem cells to lineage-restricted progenitors, progression of progenitors to postmitotic neurons and their subsequent migration to correct layer positions. The molecular mechanisms that underlie many of these early events are being unraveled. Accordingly, genetic mechanisms involving proneural genes *Ngn1/2 *and the homeodomain gene *Pax6 *have been implicated in the specification of pyramidal neurons. Similarly, proneural gene *Mash1 *and homeodomain genes *Dlx 1*, *2 *that are specific to basal ganglia are known to direct the specification of cortical interneurons [2].

In addition to the intrinsic regulators, numerous extrinsic signals that influence the cortical progenitors and neurons have been identified. Among these, chemokines are known to exert regulatory roles in the developing nervous system. Stromal derived factor (SDF-1), an alpha chemokine, has been identified in the developing and adult brain. *In vitro *studies and histological examinations have shown that SDF-1 and its receptor CXCR4 are constitutively expressed in the developing and adult cerebral cortex [3-5]. Although most chemokines are promiscuous in their ability to bind to different receptors, SDF-1 is a notable exception as it is the only known ligand that can bind to CXCR4 [6], a G-protein coupled receptor.

Interestingly, mutant mice that lack SDF-1, or its receptor CXCR4, die soon after birth with major developmental anomalies, including defects in the nervous system [7-9]. While these mutants have reduced cortical thickness the cerebellum and hippocampus show anomalous development. In the present study, using low concentrations of SDF-1, or AMD3100 in rat primary cultures we demonstrate that chemokine signaling regulates cell proliferation, at least in part through a mechanism involving intercellular communication. Further, SDF-1 enables the morphological differentiation of cortical glutamatergic neurons by inducing axon elongation and branching, and concurrently aids the neurochemical differentiation of GABAergic neurons. Finally, cortical cultures derived from CXCR4 mutants also indicate reduced cell proliferation and neuronal differentiation with fewer GABAergic neurons.

## Results

### SDF-1 maintains proliferation in primary cortical cultures

**Figure 1:**
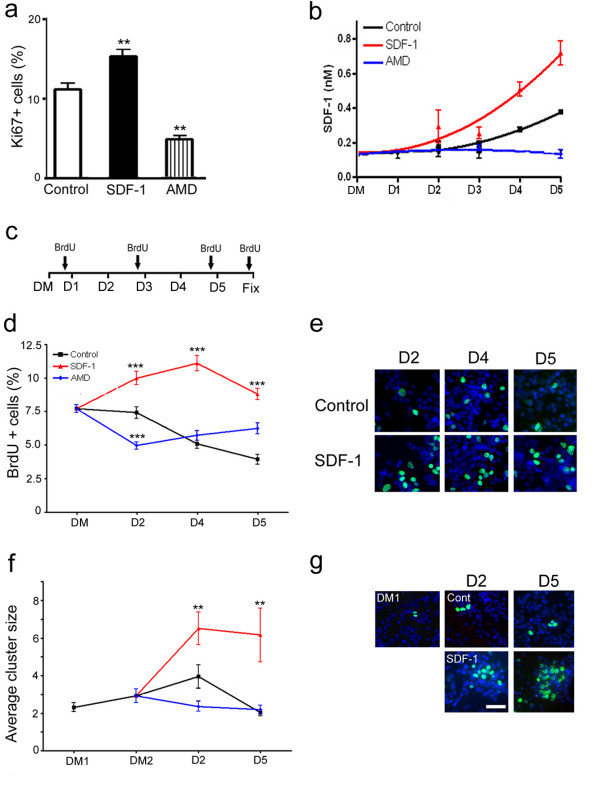
SDF-1 signaling regulates cortical cell proliferation. (a) Graph illustrates a significant increase in proliferation (Ki67+) following chronic exposure to SDF-1. (b) ELISA measurements on medium collected from control and treated cortical cultures, DM – defined medium. (c) Experimental scheme for BrdU pulsing and cluster analysis; D1–D5 corresponds to 5 consecutive days of treatment with control, SDF-1 medium or AMD3100, DM corresponds to cultures in defined medium prior to the application of any treatment. Arrows indicate the time points of BrdU applications. (d, e) Graph illustrates the rate of cell proliferation as shown by the percent of BrdU+ cells at different days of treatment, while (e) shows representative images of BrdU staining (green) against counterstained cells (blue). (f, g) Graph illustrates the average BrdU cluster size at different days of treatment and (g) shows representative images of cell clusters; DM1 and DM2 correspond to cultures in defined medium for 24 hr and 48 hr, respectively. Scale bar: 100 μm.

Stromal derived factor-1 is expressed in the developing telencephalon at a time when cell proliferation and differentiation are key events [10,5]. To investigate the role of SDF-1 signaling in cortical cell proliferation, rat E17 primary cultures were exposed to a chronic treatment of chemokine medium (4–6 nM). Subsequent examination of cultures displayed a significant increase in the number of proliferating cells, as revealed by Ki67 immunocytochemistry (Fig. 1a; *p *< 0.01, n = 4 experiments). Conversely, treatment with 40 μm of AMD3100, the receptor antagonist, reduced cell proliferation (*p *< 0.01, n = 4 experiments). Further, to determine whether chemokine delays the progenitors from exiting the cell cycle cortical cultures were pulsed with BrdU for 1 hr and examined after 5 days of treatment. Analysis of these cultures for cells that contained BrdU but were negative for Ki67 (BrdU+/Ki67-) showed the fraction of BrdU labeled cells that exited the cell cycle to become either postmitotic or quiescent. Accordingly, 91% of the BrdU labeled cells in control cultures were postmitotic compared to 81% after SDF-1 treatment (*p *< 0.01, n = 4 experiments), suggesting that the chemokine maintains the progenitors in proliferation. To ensure the presence of applied SDF-1 in culture medium, chemokine concentration was measured using ELISA after each application of treatment (Fig. 1b). Medium collected from control cultures showed the concentration of endogenous SDF-1 increasing from approximately 0.15 nM to 0.4 nM in 3 days (n = 2 experiments). However, treatment with AMD3100 appeared to prevent the baseline chemokine levels from rising above 0.15 nM, whereas application of SDF-1 medium caused a cumulative increase to a maximum concentration of 0.8 nM.

**Figure 2:**
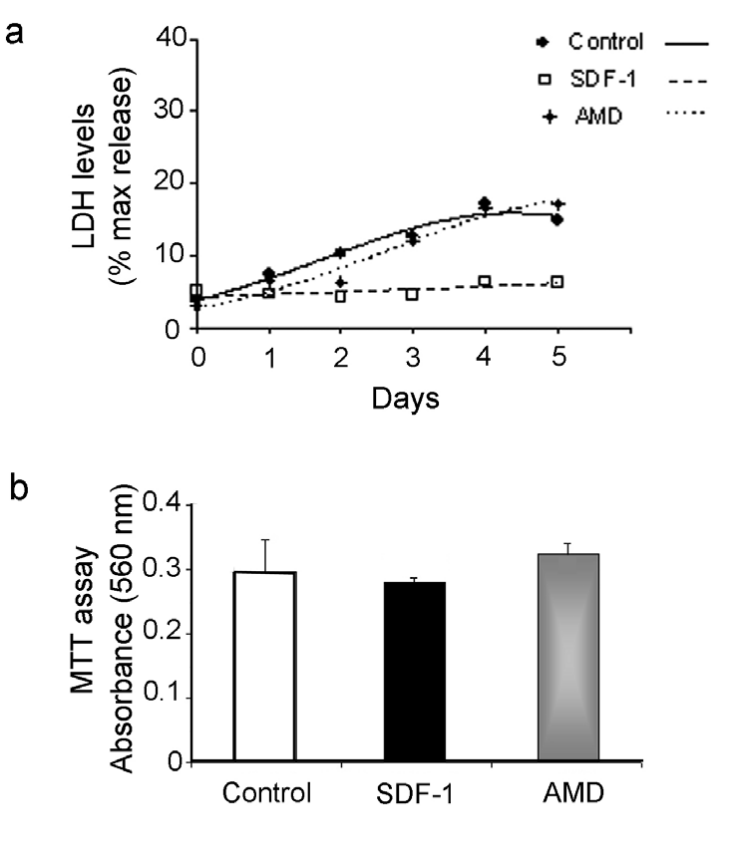
Cell death and cell survival assays. (a) Graph illustrates a representative LDH assay for measurement of cytotoxicity in medium collected from control, SDF-1 and AMD3100 treated cortical cultures; samples were assayed in triplicates for each time point. (b) Graph illustrates MTT assay for cell survival in control, SDF-1 and AMD3100 treated cortical cultures.

Given that chronic exposure to SDF-1 medium increased the progenitor population, E17 cortical cultures were pulsed with BrdU to assess the rate of proliferation at different time points of the treatment. Thus, cultures pulsed with BrdU before the application of treatment corresponding to D1, D3, D5, or 24 hr after D5 were maintained for 1 hr and fixed for analysis (see illustration in Fig. 1c). BrdU labeling of cultures grown in chemokine medium showed an increased rate of proliferation after 48 hr, which continued to remain high at both days 4 and 5 (Fig. 1d, e; *p *< 0.001, n = 3 experiments). To further illustrate the regulatory role of SDF-1 in cell proliferation, a cell cluster analysis was performed. In brief, rat E17 intact cortex was pulsed with BrdU for 1 hr prior to dissociation and the resulting cell suspension was mixed with non-BrdU labeled cells. This resulted in a culture containing clusters of BrdU+ cells in a larger population of unlabeled cells. Because BrdU is passed on to each daughter cell during mitosis the number of progeny in each cluster will provide an estimate of cell division; only those clusters that were separated by a minimum distance of 300 μm were included in the analysis. Initial analysis of cultures maintained in serum-free defined medium (DM1) showed BrdU+ clusters of 2 cells that increased to 3 after 24 hr (DM2, prior to commencing treatment). Cultures grown in control, SDF-1 medium or AMD3100 were analyzed after 2 or 5 days in treatment (Fig. 1f, g). Clusters in control cultures contained 3 cells on average, whereas exposure to SDF-1 medium markedly increased the average cluster size to 6.5 cells by day 2 (*p *< 0.01, n = 27 clusters,) and 6.2 cells by day 5 (*p *< 0.01, n = 18 clusters). We next investigated whether SDF-1 at the applied concentration promotes cell survival, or prevents cell death, which might in part explain its role in proliferation. To assess cytotoxicity, medium was collected on each day of the treatment and assayed for released LDH activity (n = 3 experiments). As shown in a representative experiment (Fig. 2a), the levels of LDH in control and AMD3100 treated cultures increased over time, suggesting a progressive increase in cytotoxicity due to cell death or stress. By contrast, the degree of toxicity in SDF-1 treated cultures remained largely unchanged over the course of the experiment. In a separate study we sought to determine if SDF-1 promotes the survival of cortical cultures (n = 3 experiments). However, MTT assays performed on end point cultures did not indicate a significant difference in survival between control and treatment groups (Fig. 2b).

### SDF-1 regulates intercellular coupling: a possible regulatory mechanism for cortical cell proliferation

It is evident from the above data that SDF-1 through its downstream signaling exerts a regulatory role in cortical cell proliferation. Earlier reports have shown that cortical progenitors express gap junctions and their proteins, connexins [11]. However, more recent studies have highlighted that connexin-mediated gap junction channels and open hemichannels in non-junctional membranes are two biologically relevant mechanisms by which progenitors can coordinate their responses to the extracellular environment [12,13]. Thus, we postulated that treatment with SDF-1 might regulate cell proliferation, at least in part through hemichannels or through docked gap junction channels in cortical progenitors. To test this hypothesis a multi-experimental approach was adopted. First, Cx 43 expression was measured in cortical cultures following exposure to SDF-1. Second, cell proliferation was assayed using Ki67 immunocytochemistry in cultures grown in SDF-1 medium in concert with carbenoxalone to block both connexin hemichannels and gap junction channels [13,14]. Third, intercellular dye-transfer was assessed in cortical explants using a modified scrape-load technique. Finally, spontaneous Ca^2+ ^transients were measured in cortical explants using real time imaging.

**Figure 3:**
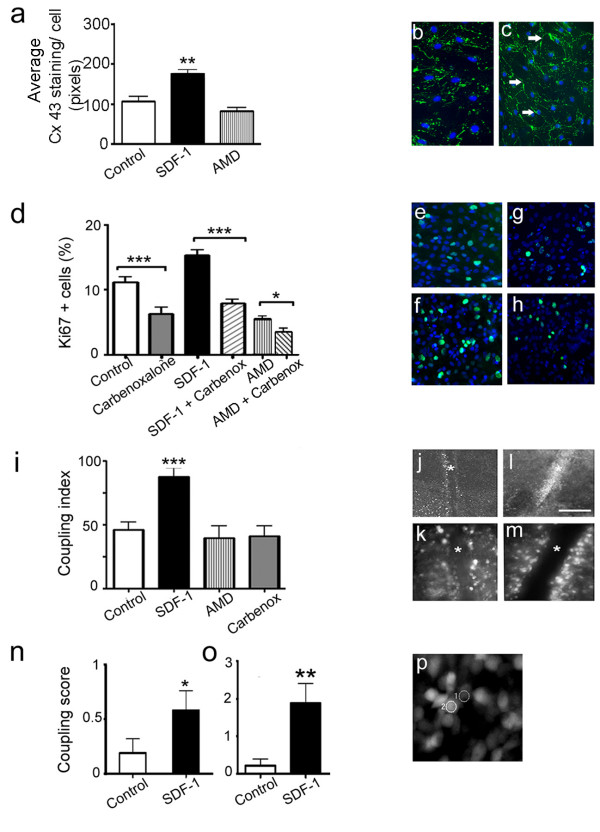
SDF-1 regulates connexin 43 expression and intercellular coupling. (a-c) Graph illustrates changes in Cx 43 immunoreactivity following SDF-1 and AMD3100 treatments in primary cortical cultures. (b, c) Representative images of control (b) and SDF-1 treated (c) cultures stained for Cx 43 (green) and DAPI counter stain (blue); arrows in (c) indicate 'plaque-like' labeling of Cx 43 immunoreactivity around cell surfaces, in contrast to the punctate staining visible in control culture. (d-h) Graph shows the percent of proliferative population (Ki67+ cells) in various treatments applied to cortical cultures. (e-h) Examples of Ki67+ cells (green) counterstained with DAPI (blue) in control (e), SDF-1 (f), carbenoxalone (g) SDF-1 + carbenoxalone (h) treated cultures. (i-m) Graph shows the extent of LY dye transfer in E17 whole mounted cortical explants that were maintained in various treatment conditions. (j-m) Examples of low and high magnification images illustrating dye transfer in control (j, k) and SDF-1 treatment (l, m), respectively; * on images indicate the scrape line. (n-p) Graphs indicate synchronized calcium transients measured in E17 whole mounted cortical explants that were in acute (n) and chronic (o) treatments. Synchronized transients were measured between adjacent cells in the cortical VZ as shown in a representative image (p). Scale bar: j, l, 250 μm; b, c, e-h, k, m, 100 μm.

To measure Cx 43 protein expression, control and treated cortical cultures were processed for quantitative immunocytochemistry. Analysis showed a marked increase in Cx 43 protein expression in cultures exposed to chemokine medium (Fig. 3a; *p *< 0.01, n = 40 fields). Interestingly, strong expression of connexin was noted outlining the cell surfaces as 'plaques' (Fig. 3c green; arrows), typical of gap junction channels; in controls (Fig. 3b) and AMD3100 treated cultures (data not shown) the pattern of staining was punctate. To determine whether SDF-1 regulates cell proliferation through connexin channels, E17 cortical cultures were treated with 10 μM carbenoxalone. Subsequent processing for Ki67 staining revealed diminished proliferation in cultures that were exposed to carbenoxalone alone (Fig. 3d, e, g*p *< 0.001, n = 35 fields), or together with SDF-1 medium (Fig. 3d, f, h*p *< 0.001, n = 22 fields). This clearly illustrates that SDF-1 mediates cell proliferation at least in part through connexin hemichannels or gap junction channels. Consistent with this finding further reduction in proliferation was noted in cultures grown together with AMD3100 and carbenoxalone (Fig. 3d).

To determine whether SDF-1 treatment alters intercellular dye transfer to reflect gap junction channel coupling a modified scrape-load technique was used. Briefly, cortical explants that were whole mounted with ventricular surface on the top were scrape-loaded with a mixture of LY and rhodamine dextran. The extent of LY transfer was then quantified into a coupling index (Fig. 3i–m). Remarkably, explants that were maintained in SDF-1 medium showed a 2-fold increase in dye-transfer compared to controls (Fig. 3j–m, *p *< 0.001, n = 15 explants). Finally, to measure the spontaneous Ca^2+ ^transients in VZ cells, cortical explants exposed to either acute (15 min) or chronic (24 hr) SDF-1 treatment were loaded with Oregon Green BAPTA, a Ca^2+ ^indicator and analyzed using real time imaging. Images were collected every second and changes in the average intensity were converted into a 'cell coupling score' [15]. Interestingly, exposure of cortical explants to either acute or chronic chemokine treatment significantly increased the frequency of synchronized Ca^2+ ^transients between adjacent VZ cells (acute treatment: Fig. 3n, *p *< 0.05, n = 19 pairs of cells; chronic treatment: Fig. 3o, *p *< 0.01, n = 33 pairs of cells). To confirm that the coupling was indeed in the cortical VZ, explants were subsequently fixed and processed for Ki67 immunocytochemistry (data not shown). Taken together, these results illustrate that SDF-1 signaling regulates intercellular coupling and Cx 43 protein expression in cortical progenitors. Although, it is very likely that the coupled progenitors express Cx 43 the contribution of other connexins cannot be ruled out.

### SDF-1 signaling regulates the differentiation of cortical neurons

**Figure 4:**
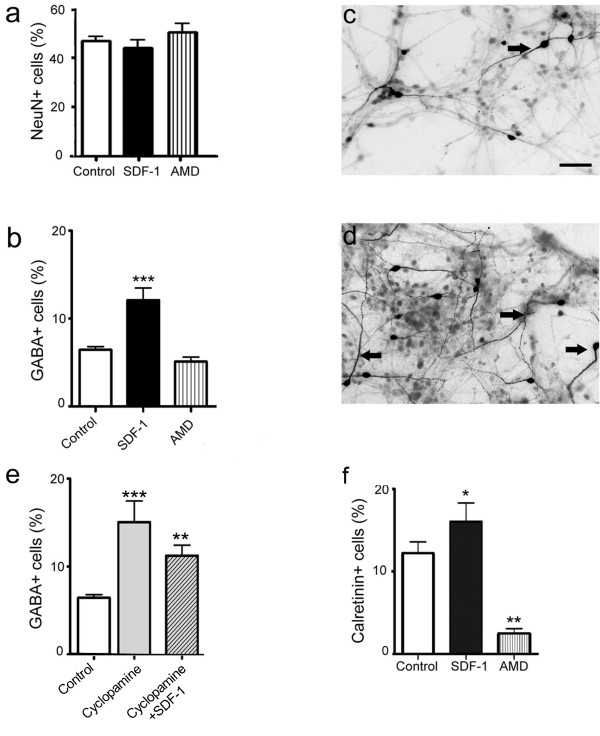
SDF-1 regulates the differentiation of cortical neurons. (a) Graph shows the percent of postmitotic neurons (Neu-N + cells) in control and treated cortical cultures. (b-d) Graph illustrates the percent of GABA+ neurons in various treatments applied to cortical cultures (b). Examples of images from control (c) and SDF-1 treated (d) cultures stained for GABA immunoreactivity; arrows indicate labeled cells. (e) Treatment with cyclopamine (Shh antagonist) alone or together with SDF-1 increased the GABA expression, suggesting a sonic independent mechanism. (f) Graph shows the percent of calretinin immunoreactivity in control and treated cultures. Scale bar: 100 μm.

To investigate the role of SDF-1 signaling in regulating neuronal differentiation, rat E17 cortical cultures were immunolabeled for Neu-N, a neuron specific marker. Analysis did not indicate a significant difference in the output of neurons between control and treatment groups (Fig. 4a), suggesting that the chemokine does not affect the differentiation of glutamatergic neurons, which constitute 80–85% of all cortical neurons as has been shown in both *in vivo *and in *vitro *studies [16-18]. Next, to examine whether SDF-1 signaling regulates the differentiation of cortical GABAergic neurons cultures were processed for GABA immunocytochemistry. Interestingly, a 2-fold increase in GABA+ neurons was noted in cultures grown in SDF-1 medium compared to controls (Fig. 4b–d, *p *< 0.001, n = 47 fields). Recent studies have implicated a role for Shh signaling in the differentiation of cortical GABAergic neurons [19]. As SDF-1 has been shown to modulate Shh signaling in the developing cerebellum [20] we reasoned that a similar mechanism might regulate the differentiation of cortical GABAergic neurons. Thus, cortical cultures were exposed to cyclopamine, a Shh antagonist [19], alone or in the presence of SDF-1 medium. Although, application of cyclopamine to control cultures did not show a marked difference in the output of neurons as indicated by Neu-N staining, an increase was noted in the expression of GABA+ neurons (Fig. 4e, *p *< 0.001, n = 23 fields). Further, cultures grown in SDF-1 medium together with cyclopamine also showed elevated GABA expression, similar to the response noted with either of the treatments alone (Fig. 4e, *p *< 0.01, n = 33 fields).

We next asked whether the increase in GABA expression would be reflected in the early subtypes of cortical interneurons. Thus, E17 cortical cultures were examined for calretinin, an early interneuronal subtype, following treatment with chemokine or antagonist. Remarkably, application of chemokine significantly increased the calretinin+ neurons (Fig. 4f, *p *< 0.05, n = 20 fields), whereas the antagonist reduced the expression by 75%.

### SDF-1 signaling regulates axon growth and branching in cortical neurons

**Figure 5:**
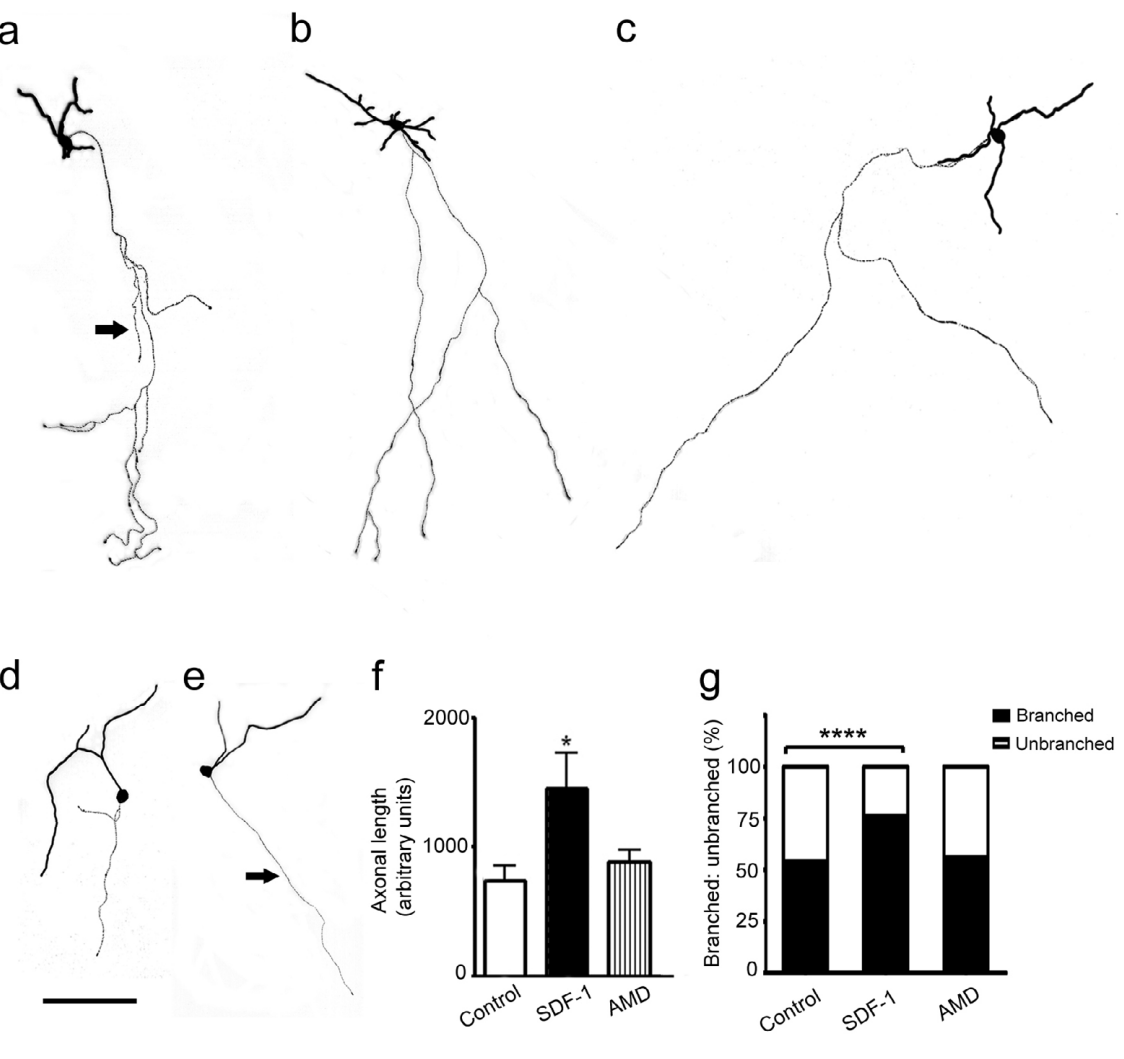
SDF-1 promotes axon elongation and branching in cortical neurons. (a-f) Representative reconstructions of GFP labeled neurons in SDF-1 treated (a-c) and control (d, e) cultures; note the elongated and branched axons in SDF-1 treatment compared to shorter axons in controls (arrows). (f) Graph illustrates the average length of GFP+ axons in control and treated cultures. (g) Stacked graph shows the frequency of branching in control and treated cultures. Scale bar: 90 μm. ****: Significance at p < 0.0001 (chi-square test).

**Figure 6:**
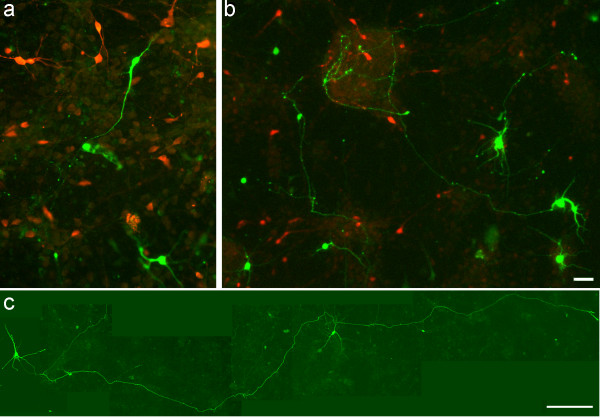
Characterization of GFP labeled neurons in cortical cultures. (a, b) Representative images of GFP transfected control (a) and SDF-1 treated (b) cultures labeled for GABA immunoreactivity (red staining). (c) Image of a GFP+ neuron with an extended axon in SDF-1 treated culture. Scale bar: a, b, 30 μm; c, 150 μm.

Stromal derived factor-1 signaling has been shown to regulate axon outgrowth in the developing cerebellum and hippocampus [21,22]. Thus, to examine whether the chemokine exerts a similar regulatory mechanism in the developing cortex, E17 cultures were transfected with GFP to randomly label cells. A transfection efficiency of 20–25 GFP+ cells/coverslip was chosen for the morphological analysis of neurite outgrowth. Following transfection, cortical cultures were maintained in control, SDF-1 medium or AMD3100 as described previously. Although, the longest neurite was defined as an axon [22], immunocytochemistry was performed to label neurofilaments that are specific to axons (data not shown). Quantitative analysis confirmed that GFP+ neurons grown in SDF-1 medium showed a greater degree of morphological differentiation with elongated axons compared to those grown in control conditions (Fig. 5a–f, *p *< 0.05, n = 45 cells). Further, SDF-1 increased the frequency of axon branching, however the treatment did not alter the number of branches, at least at the low concentrations of chemokine used (Fig. 5g, *p *< 0.001, chi-square test, n = 45). To characterize the phenotype of GFP+ neurons, control and treated cultures were labeled for GABA immunoreactivity. Interestingly, 95% of GFP+ neurons in both groups were GABA negative, thus confirming their glutamatergic origin (Fig. 6a, b). Further, cortical glutamatergic pyramidal neurons are known to have extended axons; this also alludes to the phenotype of GFP+ neurons in treated cultures as glutamatergic neurons (Fig. 6c).

### Proliferation and neuronal differentiation are altered in CXCR4-/- mutant cortical cultures

**Figure 7:**
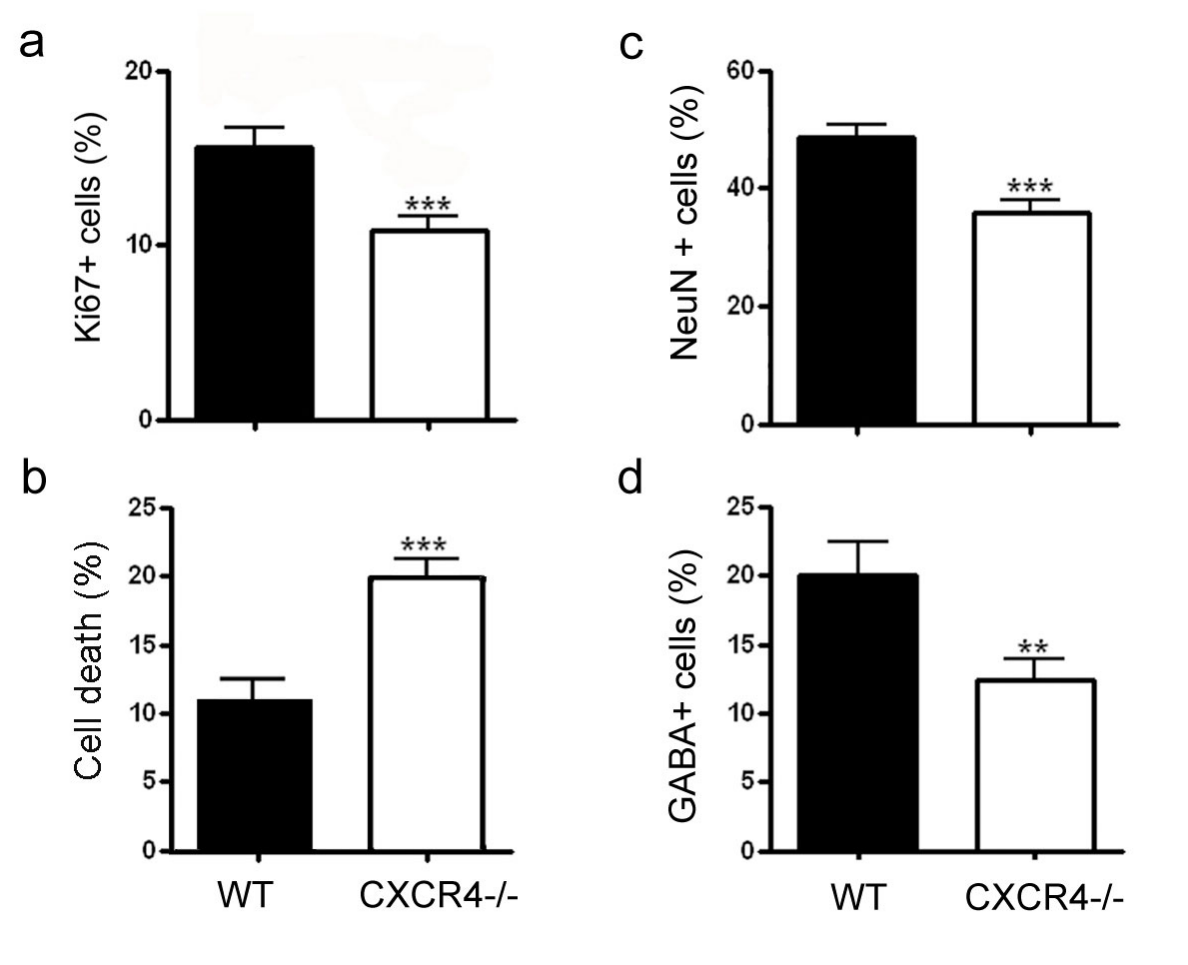
Proliferation and differentiation are reduced in E15 CXCR4-/- cortical cultures. (a, b) Graphs show diminished proliferation as evidenced by Ki67 immunolabeling (a) and increased cell death quantified by propidium iodide or trypan blue staining (b) in mutant cultures compared to wild type controls. (c, d) Staining of cultures for Neu-N also showed fewer postmitotic neurons (c) and GABAergic neurons (d) in mutant cultures compared to wild type controls.

In agreement with the data from antagonist treatment, examination of E15 cortical cultures derived from CXCR4-/- mutant mice showed diminished proliferation as evidenced by Ki67 immunocytochemistry (Fig. 7a, *p *< 0.001, n = 47 fields from 9 mutants). Further, estimation of mitotic profiles confirmed reduced mitotic activity in CXCR4-/- cultures compared to wild type controls (data not shown). Screening of cultures stained for propidium iodide or trypan blue indicated a marked increase in cell death in mutant cultures compared to wild type (Fig. 7b, *p *< 0.001, n = 47 fields from 9 mutants). To assess neuronal differentiation, cultures were processed for Neu-N and GABA immunocytochemistry. Analysis of mutant cultures revealed diminished output in both the total neurons (Fig. 7c, *p *< 0.001, n = 68 fields) and GABAergic population compared to wild type controls (Fig. 7d, *p *< 0.01, n = 45 fields). Previous studies have indicated reduced cortical thickness in CXCR4-/- mutants with no gross defect. Consistent with the earlier findings, staining of E15 mutant brain sections (n = 3 mutants) for Ki67 indicated a mild anomaly in the proliferative population [see Additional file 1]. Line-scan analysis of immunolabeled sections (n = 5 representative sections/brain) showed reduced pixel intensity (approximately 20%) in mutant cortex compared to wild type [see Additional file 1]. Although, examination of wild type and mutant brain sections did not show a notable difference in neuronal staining [see Additional file 1], labeling for GABA displayed an altered pattern of immunoreactivity. Typically, GABAergic neurons that originate in the ventral telencephalon migrate tangentially through the subventricular zone, intermediate zone and marginal zone [23,24]. However, in the mutants the marginal zone contained distinctly fewer GABA+ neurons while the intermediate zone appeared to have increased immunoreactivity compared to those in wild type tissue [see Additional file 1]. Similar pattern of staining was observed with calbindin, also a marker of migrating cortical GABAergic neurons (data not shown).

### SDF-1 signaling regulates down stream gene expression

**Figure 8:**
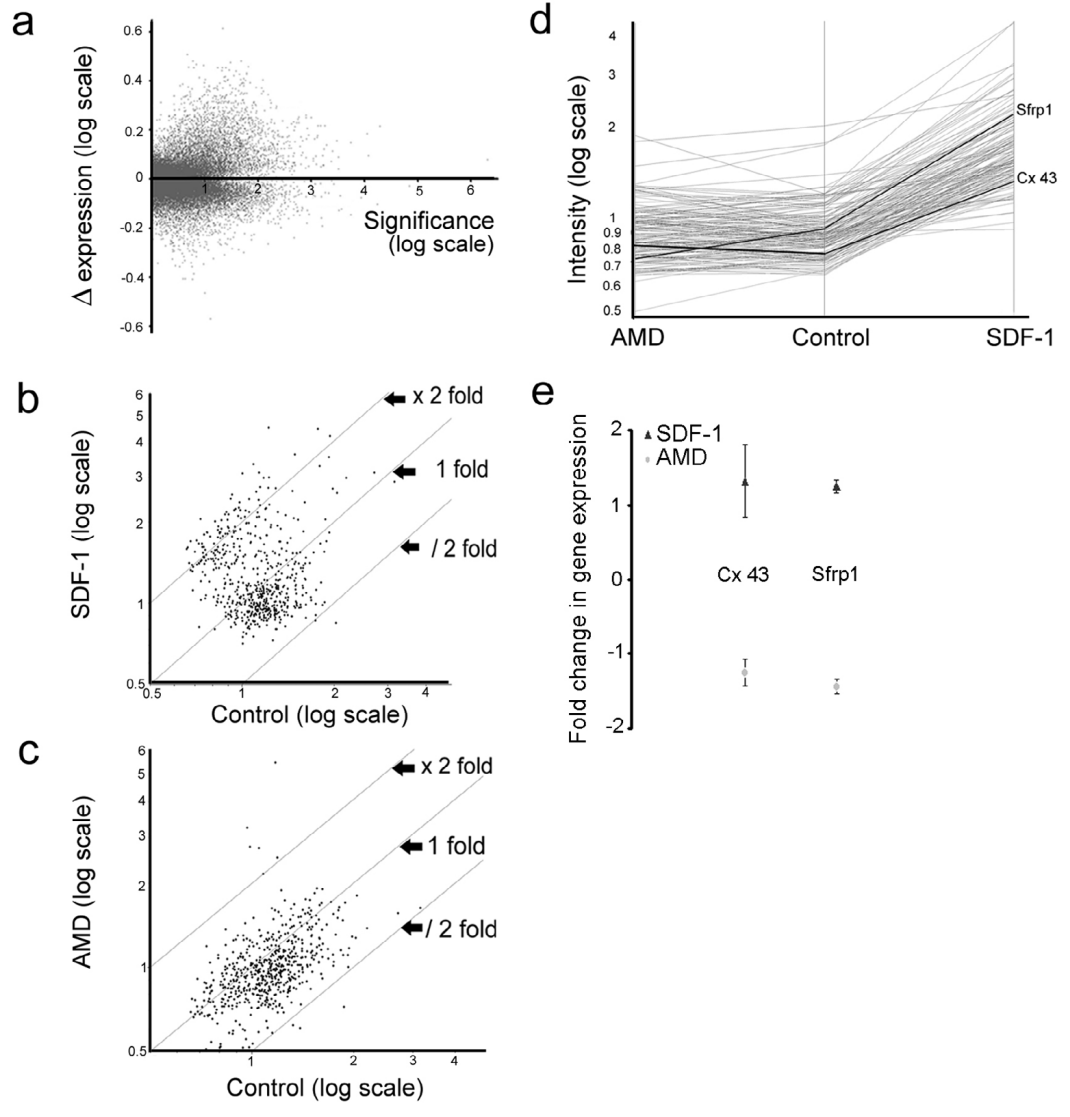
SDF-1 signaling activates downstream genes. (a-d) Illustrates data from Affymetrix rat genome RG-230v2 microarrays analysis; every data point represents the average of 3 independent hybridizations (biological replicates) per experimental condition. (a) Illustrates the change in the mean expression level of all hybridized transcripts between control and SDF – treatment. (b, c) Illustrates transcripts that showed a near 2-fold (37%) or greater than 2-fold (12%) up regulation in SDF-1 treatment (b), and a near 2-fold (72%) down regulation in AMD3100 treatment (c) compared to controls. (d) Cluster analysis shows genes of biological significance being grouped together (e) qRT-PCR analysis to validate the gene expression of *Cx 43 *and *Sfrp1*.

To identify the differentially expressed transcripts following chemokine treatment, total RNA was extracted from rat E17 cortical cultures grown in control, SDF-1 medium or AMD3100; three biological replicates were used for each experimental condition. The synthesized cRNA was labeled and hybridized to Affymetrix rat genome RG-230v2 microarrays according to the manufactures guidelines. Following background correction and normalization, the intensities of the biological replicates were averaged to obtain the mean expression value for each transcript in all experimental conditions. Thus, every data point in figures 8a–d represents the average of three independent hybridizations (biological replicates). Figure 8a illustrates the change in the mean expression level of all hybridized transcripts (31,042 probes). The dataset was then filtered using a threshold for expression level and fold change to generate a list of 584 transcripts. Of these, 12% of the transcripts showed an upregulated expression greater than 2-fold following treatment with SDF-1, while 37% displayed an intermediate expression that was less than 2-fold (transcripts between fold lines x2 and x1; Fig. 8b). By contrast, 72% of the probes showed a near 2-fold down regulation in response to AMD3100 treatment (transcripts between fold lines/2 and x1; Fig. 8c). The filtered data was further processed using K-means with Pearson correlation and pre-specified number of clusters to group transcripts into sets of clusters. This analysis yielded a cluster of 142 transcripts that showed an upregulated response to SDF-1 and conversely down regulated in the presence of AMD3100 (Fig. 8d). Among these, *Cx 43 *and secreted frizzled related protein 1 (*Sfrp1*) were of direct relevance for the regulatory functions of SDF-1 in cell proliferation and axon growth, respectively. These two transcripts showed a marked upregulation of 1.9-fold and 2.4-fold in response to SDF-1 treatment, respectively. Subsequent validation with qRT-PCR confirmed that *Cx 43 *and *Sfrp1 *were indeed upregulated by SDF-1, although the magnitude of fold change was less than that observed in microarray analysis (Fig. 8e).

## Discussion

### SDF-1 signaling maintains cortical progenitors in proliferation by facilitating intercellular coupling

Treatment of rat E17 cortical cultures with SDF-1 medium showed a marked increase in proliferation as evidenced by Ki67 immunocytochemistry and BrdU labeling techniques. The increase in the number of BrdU+ cells in defined medium suggests a cell cycle length of 20–24 hr consistent with earlier reports [25]. Our experiments indicate that the progenitors are likely to have divided asymmetrically [26] to yield 3- cell clusters in defined medium, which then continued to generate clusters of 6.5 cells after 48 hr in SDF-1 treatment. In support of this view, quantitative estimation of cells that were BrdU+/Ki67- suggests that SDF-1 impedes the progenitors from exiting the cell cycle. By contrast, the proportion of dividing cells in control cultures was less, as many would have exited the cell cycle to become either postmitotic or quiescent. Taken together, it is evident that SDF-1, at concentrations less than 10 nM, maintains the cortical progenitors in proliferation and possibly enables their survival by reducing cytotoxicity. However, it is less likely for the treatment to have shortened the cell cycle time, or switched the mode of cell division from asymmetric to symmetric division. It is pertinent to note that in the proliferation of cerebellar granule cells the mitogenic potency of SDF-1 is only apparent in concert with Shh, a potent mitogen expressed by Purkinje neurons [20]. Interestingly, a recent study has highlighted that SDF-1 induces the proliferation of cortical neurospheres that were expanded in basic fibroblast growth factor (bFGF), a known mitogen [27]. Remarkably, the mitogenic effect of SDF-1 noted in our preparations has close parallels to the activity of bFGF in rat cortical cultures [28]. Accordingly, the growth factor maintains the progenitors in cycle by increasing the number of cell divisions, stimulates the quiescent cells into proliferation and enables their survival without shortening the length of cell cycle. Although, a direct link between SDF-1 and bFGF is yet to be shown, it is more likely that both factors share common downstream signaling mechanisms that facilitate the proliferation of cortical progenitors. Notably, ERK1/2 and PI-3 kinase pathways have been implicated in the SDF-1 induced proliferation of bFGF- expanded neurospheres [27]. In agreement with the data from antagonist experiments in rat cultures, examination of CXCR4-/- cultures showed diminished mitotic activity and increased cell death. Further, staining of mutant brain sections for Ki67 immunoreactivity revealed a minor anomaly in cell proliferation consistent with the earlier finding of reduced cortical thickness [29].

Earlier studies have demonstrated that cortical progenitors are coupled by a network of gap junction channels that regulate the entry of dividing cells into S-phase [30,11,31]. However, it is now evident that undocked connexin hemichannels distributed in the progenitors during late G_1 _or S-phase propagate spontaneous Ca^2+ ^transients that may modulate proliferation [13]. In our experiments treatment with SDF-1 significantly increased the cortical progenitor population (Ki67+ cells), which declined sharply when exposed to carbenoxalone, a blocker of both connexin hemichannels and gap junction channels. Further, treatment of cortical cultures with chemokine medium increased Cx 43 immunoreactivity with a pattern of staining that was robust and predominantly localized to the cell surface. Thus, to distinguish whether chemokine treatment influences the undocked connexin hemichannels or gap junction channels, LY dye transfer experiments were performed in whole mounted cortical explants. Interestingly, the scrape-load technique clearly illustrated that in the presence of SDF-1 medium the degree of dye transfer was significantly more on both sides of the scrape line compared to control explants. Although, a few cells at the ventricular surface were filled with LY, possibly through open hemichannels, they were located away from the scrape line in both control and SDF-1 treated explants. Open hemichannels are known to be prevalent in lower Ca^2+ ^conditions [14], thus few undocked channels may have remained open in our preparations as they were maintained in regular Ca^2+ ^containing medium. In this context, our calcium imaging experiments corroborate the LY dye transfer data. As Ca^2+ ^transients were recorded from cells that were positioned within a depth of three layers from the VZ surface, it is very likely these cells were in late G_2_, M or early G_1 _phase of the cell cycle. Based on the premise that connexin hemichannels are distributed on the membranes of progenitors in late G_1 _or S-phase [13], the spontaneous Ca^2+ ^transients recorded in our experiments are likely to have propagated through gap junction channels.

### SDF-1 mediated differentiation of cortical neurons

Our experiments showed that SDF-1 treatment significantly up regulates the differentiation of GABAergic neurons, including the calretinin subtype. Although chemokine treatment has been shown to promote the survival of neurons in other systems [32], we believe that alone cannot increase the differentiation of GABAergic neurons. A growing body of evidence now suggests that cortical progenitors do have the competency to generate GABAergic neurons *in vitro *[16,19,33].

Interestingly, while the work of Gulacsi and Lillien showed that the balance between the inductive and repressive signals of Shh and BMP, respectively, determines the expression of GABA+ neurons, Nery and colleagues have illustrated that cortical progenitors are competent to express *Dlx 2 in vitro*. In light of these earlier reports, it is plausible for the chemokine signaling to have induced the generation of cortical GABAergic neurons *in vitro*. However, staining of control and treated cultures for BrdU and GABA immunocytochemistry did not yield a quantifiable co-labeling to suggest that GABAergic neurons were generated *in vitro *(data not shown). Thus, it is conceivable that SDF-1 induced the expression of GABA in postmitotic neurons that were yet to assume a neurotransmitter identity. Alternatively, a subset of cortical glutamatergic neurons may have transiently expressed GABA in response to chemokine treatment. Although, our experiments illustrated an increase in GABA+ neurons in response to SDF-1, it is not known whether these cells function as inhibitory interneurons, or would continue to express GABA in the absence of chemokine signaling. Further, the effects of SDF-1 and antagonist treatment were notably evident in the cortical calretinin population. Calretinin, a marker of preplate cells during early development is later expressed in a subset of GABAergic neurons. Because our rat cultures were derived from embryos of mid corticogenesis stage, it is less likely for the chemokine treatment to have induced the differentiation of preplate neurons. Moreover, the majority of calretinin+ neurons in our cultures appeared bipolar in shape, suggesting a GABAergic lineage in contrast to the preplate cells that exhibit multipolar morphology *in vitro *[34]. Interestingly, cortical cultures derived from CXCR4-/- mice also showed diminished neuronal differentiation and importantly fewer GABAergic neurons. Consistent with these culture experiments, examination of E15 mutant brain sections showed distinctly fewer GABA+ neurons in the marginal zone compared to wild type tissue. It is possible that loss of chemokine signaling may have dispersed or misdirected the movement of a subset of neurons from the marginal zone towards the intermediate zone, as an increase in GABA immunoreactivity was noted in the latter region. In this context, it is pertinent to note that secreted SDF-1 in the meninges enhances the dispersion of a subset of Cajal-Retzius cells derived from cortical hem and that loss of chemokine activity causes disorganization of these cells [35,36]. Sonic hedgehog signaling has been implicated in the generation of cortical GABAergic neurons [19]. However, in our experiments blocking of Shh signaling with cyclopamine increased the output of GABA+ neurons alone or in concert with SDF-1. This suggests, unlike in cerebellum [20], SDF-1 signaling does not interact synergistically with Shh, at least for the differentiation of GABAergic neurons.

A noteworthy finding was the elongation of axons of cortical projection neurons in cultures exposed to chemokine medium. In addition, neurons that showed axon branching with one or more collaterals were prevalent in cultures grown in SDF-1 medium. Similar findings have been reported in cultured cerebellar granule neurons [21] and hippocampal neurons [22]. Accordingly, both groups of neurons responded differently to SDF-1 treatment depending largely on the applied dosage; lower concentrations of chemokine induced axon elongation in cerebellar granule neurons with apparently no change in the number of branches, while higher concentrations abolished the effects. It is relevant to note that the concentration of chemokine used in our experiments was much lower and closer to the physiological limits than those used in earlier studies. What might be the significance of SDF-1 mediated regulation of cortical axons? A recent study has highlighted that SDF-1 through its G-protein coupled receptor modulates the repellent activities of Slit-2 in cultured retinal ganglion cell axons [32]. Given that Slit-1, a repellent of cortical axons [37], closely follows the endogenous expression of SDF-1 in the developing cortex raises the possibility that the chemokine might modulate the repressive signals and provide a permissive environment for the cortical afferents.

### Down stream gene activation of SDF-1 signaling

Using gene chip analysis together with qRT-PCR, we have explored the activation of downstream genes following chemokine treatment in cortical cultures. Among the genes of interest that were identified, *Cx 43 *showed a 2-fold rise following SDF-1 treatment thereby validating the findings of increased protein expression and intercellular coupling. Another gene of relevance to the regulatory function of SDF-1 is *Sfrp1*, which showed an upregulated expression greater than 2-fold. Interestingly, *Sfrp1*, expressed in the developing cortical VZ [38], has been shown to promote neurite outgrowth in retinal neurons *in vitro *[39]. Thus, it is entirely plausible that elevated *Sfrp1 *may have contributed to the elongation of axons as visualized in our cortical cultures. In addition, the upregulation of *MAP1b*, *Ncam1 *and *MAP kinases 1 and 9 *that are components of many downstream signaling pathways may have directly or indirectly contributed to the regulatory functions of SDF-1 in cortical cultures.

## Conclusion

Using cellular, molecular and functional assays the present study demonstrates that SDF-1 signaling exerts multiple regulatory roles on distinct cortical cell populations *in vitro*. Our results demonstrate that exogenous SDF-1, at low concentrations, can exert a mitogenic effect by retaining the cortical progenitors in proliferation possibly through enhanced intercellular communication. Concurrent to its role in proliferation, chemokine signaling regulates the morphological differentiation of cortical glutamatergic neurons and enables the neurochemical differentiation of GABAergic neurons. The existing CXCR4-/- and SDF-1-/- mutants do not provide a clear phenotype in the cortex, possibly due to compensatory mechanisms. In the absence of adequate genetic approaches to elucidate the *in vivo *significance of SDF-1 and CXCR4, the emerging *in vitro *and *ex vivo *studies indicate that the chemokine signaling plays an important regulatory role in the developing cerebral cortex and the nervous system at large.

## Methods

### Preparation of SDF-1 conditioned medium

Confluent HEK293 cells, cultured in the presence of DMEM: F12 (Sigma), 10% fetal bovine serum, 100 μM L-glutamine, 5 U/ml penicillin and 5 mg/ml streptomycin in a humidified 5% CO_2 _incubator at 37°C, were transfected with rat SDF-1α cDNA (GenBank AF209976 amplified from rat cDNA library) that had been sub cloned into a pcDNA3 vector (mouse and rat SDF-1α amino acid sequences share 97% identity). For controls, mock transfections were prepared using DNA-free transfection solution. Supernatant was collected after 48 hr from SDF-1 transfected (SDF-1 medium) and untransfected cultures (control medium) and stored at -20°C for future application.

### ELISA

The concentration of rat SDF-1 was measured by enzyme linked immunosorbent assay with modifications to a commercially available kit (Quantikine human SDF-1α; R&D Systems). Briefly, 50–100 μl of culture medium or standards of known concentration of SDF-1 were added to each well of 96-well microplates, pre-coated with mouse monoclonal antibody to SDF-1α. The plates were incubated at room temperature for 2–3 hr, washed (×4) with wash buffer and treated with 200 μl/well of horseradish peroxidase-conjugated polyclonal antibody to SDF-1α. The plates were further incubated for 2–3 hr, washed (×4) and treated with 200 μl/well of freshly prepared tetramethylbenzidine in hydrogen peroxide and kept in the dark for 45 min. Reaction was then arrested with 50 μl/well of 2 M sulphuric acid, after which the absorbance was measured at 450 nm using a micro plate reader.

### Primary neural cell cultures

Cortices (excluding hippocampus) were isolated from rat E17 brains and dissociated as described previously [40]. Cells, plated at a density of 1.5 × 10^5 ^on poly-L-Lysine/laminin (10 μg/ml) coated glass coverslips, were maintained for 24 hr in 10% serum containing medium and thereafter transferred to serum-free defined medium supplemented with 1 × N2 (Invitrogen). To test the effect of chemokine, serum-free cultures were treated with chemokine medium (4–6 nM SDF-1) or grown in control medium (mock transfected HEK cell medium). Likewise, to block receptor activity, serum-free cultures were grown in 40 μM of AMD3100 (NIH), dissolved in DMSO (final concentration 1:1000). To verify the cytotoxicity of DMSO, pilot experiments were performed with appropriate vehicle controls. To block gap junction coupling, cultures were exposed to 1 μM carbenoxalone, or to inhibit sonic hedgehog signaling 5 μM cyclopamine was used. Once in serum-free defined medium, cultures were treated with control, chemokine medium or AMD3100 every 24 hr for 5 consecutive days and fixed for immunocytochemistry.

To assay cell proliferation on different days of the experiment, cultures were pulsed with 30 μM BrdU for 1 hr before the application of treatment appropriate for the day. To visualize and quantify neuronal processes, primary cultures grown in either control or treatment medium were transfected with GFP construct using Lipofectamine 2000.

### CXCR4 -/- mice

B6.129X-Cxcr4tm1Qma/J mice were obtained from Jackson Laboratory (Maine, USA). Genotyping of tissue was carried out as described previously [41]. Primary neural cultures were prepared from E15 wild type and mutant cortex as described above. Once in serum free conditions, cultures were further maintained for 5 days in defined medium supplemented with 1 × N2 and fixed for immunocytochemistry. To examine the pattern of immunostaining of Ki67, MAP2, GABA and calbindin in brain sections, E15 wild type and mutant brains were fixed in 4% paraformaldehyde/0.1 M phosphate buffer and sectioned using a cryostat. Coronal sections were processed for immunocytochemistry as described below.

### LDH and MTT assay

Medium collected from control and treated cultures every 24 hr was assayed for LDH activity to obtain a measure of cytotoxicity. The assay is based on the conversion of tetrazolium salt into a red formazan product by LDH released from lysed cells. The intensity of color, proportional to the degree of cytotoxicity, was quantified by measuring absorbance at 492 nm (n = 3 experiments). To assay cell survival, control and treated cultures were incubated with MTT for 2 hr at the end of the experiment (end point cultures). Medium containing purple formazan crystals, formed by active mitochondrial enzymes, was dissolved in DMSO and the intensity measured at 560 nm (n = 3 experiments).

### Preparation of cortical explants

Cortical explants were prepared from rat E17 brains. Briefly, embryonic cortices dissected free from the adjacent hippocampus and underlying eminence were flat-mounted onto 0.45 μm nitrocellulose filters with VZ surface on the top. The explants were then maintained in control or treatment medium for 1–2 days.

### Immunocytochemistry

Primary cells were fixed in 4% paraformaldehyde/0.1 M phosphate buffer (pH 7.4) and processed for immunocytochemistry. Cells were incubated overnight with primary antibodies for BrdU (mouse monoclonal, 1: 300, Progen), GABA (rabbit polyclonal, 1:750, Sigma), calbindin (rabbit polyclonal, 1:750, Swant), calretinin (rabbit polyclonal, 1:750, Swant), NeuN (mouse monoclonal, 1: 400, Chemicon), MAP2 (mouse monoclonal, 1:500, Sigma), Ki67 (rabbit polyclonal, 1:750, Chemicon) or connexin 43 (1: 100, Zymed). After washing, cells were incubated with FITC or rhodamine-conjugated secondary antibodies (mouse or rabbit, 1:500, Molecular Probes,) at room temperature for 2 hr. Labeled cells were counterstained with DAPI and examined with a Nikon EC600 fluorescent microscope equipped with a cooled CCD system. Sequential images were subsequently reconstructed using Metamorph imaging software.

### Image analysis

To measure the relative abundance of Cx 43 immunoreactivity a modified method was adopted [42]. All images were analyzed using Metamorph imaging software. Briefly, fluorescent images collected from control and treated samples were initially processed for background subtraction followed by removal of single pixels using median filter. Based on the pixel intensities, which ranged from 0–255, Cx 43 signal was distinguished from the background by empirically determining an intensity value of 80. Images were then thresholded and the fluorescent signal was measured using the automated function of the software. Total cell count per image was obtained by counting DAPI stained nuclei. Because of the varying degree of protein expression, the fluorescent signal is presented as 'average Cx 43 expression per cell'.

### Calcium imaging

E17 cortical explants that were maintained in either control or treatment medium for 1–2 days were loaded with Oregon Green BAPTA for 20 min (10 μM/ml; Molecular probes) and transferred to a temperature controlled glass chamber (35–37°C) for image acquisition. Images were collected every second for at least 3–5 min and subsequently analyzed using Metamorph imaging software. Change in fluorescent intensity was converted into a coupling score. A coupling event was defined as a 10% or greater change in fluorescent intensity between pairs of neighboring cells [15].

### Dye transfer in cortical explants: scrape load method

To test for dye coupling, rat E17 cortical explants that were maintained in either control or treatment medium for 1–2 days were scrape loaded [43]. Briefly, a sharp incision was made across the explants and loaded with a mixture of 1% Lucifer yellow/rhodamine dextran for 20 min. Explants were then fixed and images were acquired for analysis. To ensure that the explants were mounted with the VZ surface on the top, they were processed for Ki67 immunocytochemistry to label proliferative cells. The degree of dye transfer was converted into a coupling index defined by the ratio between LY labeled area and the length of corresponding cortical explant.

### DNA microarrays

Briefly, rat E17 control and treated cultures (litter of embryos/biological replicate) were homogenized in trizol. The RNA precipitated from the aqueous phase by the addition of isopropanol was washed, air dried and quantified. Microarrays were used as described in the Affymetrix gene chip technical manual. Briefly, 15 μg of total RNA was converted to cDNA followed by in vitro transcription for linear amplification of transcripts and incorporation of biotinylated CTP and UTP. The cRNA products were fragmented to 200 nucleotides or less, labeled and hybridized for 16 hr at 45°C to Affymetrix rat genome RG-230v2 microarrays. The arrays were washed at low and high stringency before staining with streptavidin-phycoerythrin. Fluorescence was amplified using biotinylated anti-streptavidin followed by incubation with streptavidin-phycoerythrin stain. Technical quality control was performed with dChip (V2005) using the default settings. Background correction and quantile normalization were performed using RMA Express [44]. Normalized data was filtered first on expression intensity and then by fold change in expression using GeneSpring software (Agilent Technology). Genes of interest were subsequently validated with qRT-PCR.

### Quantitative RT-PCR

Using real-time quantitative PCR method, the relative quantity of transcripts was measured in control and treated samples. All genes of interest were first normalized with GAPDH and β-actin, the house keeping genes. The relative abundance of transcripts in treated samples (SDF-1 and AMD3100) was then expressed as fold change compared to those measured in controls. To ensure there was no DNA contamination, RNA samples were first treated with DNase. cDNA was then generated by reverse transcription: briefly, samples were incubated with 25 ng/μl random primers and 0.77 mM dNTPs for 5 min at 65°C. After 1 min on ice, samples were centrifuged briefly and incubated with 50 mM Tris-HCl, 75 mM KCl, 3 mM MgCl_2_, 5 mM DTT, 2 units RNase inhibitor, and 10 units reverse transcriptase for 5 min at 25°C. For reverse transcription the mixture was left at 50°C for 45 min and the reaction inactivated by heating to 70°C for 15 min. The prepared cDNA was stored at -20°C. Taqman qRT-PCR [45] was performed in 96-well plates; cDNA (5 μl per well) was added to 10 μl 2× master mix to yield 5 mM MgCl_2 _containing uracil-N-glycosylase (UNG), 0.8 μM of relevant primer and 0.4 μM of relevant probe. PCR reactions and analysis were carried out using an ABI Prism 7700 Sequence Detector. Briefly, 50°C for 2 min, 95°C for 10 min followed by 35–40 cycles of 92°C for 15 sec and 60°C for 60 sec. Primer and probes were designed and chosen using the Roche Applied Science Probe Finder software v2.10.

### Statistical analysis

To determine the level of significance between control and various treatments, analysis of variance (ANOVA) was used with Bonferroni's posttest correction. To compare the significance between two parametric variables, Student's unpaired t-test was performed while for the analysis of categorical variables (branched or unbranched axons) chi-square analysis was used. Error bar in graphs represent standard error of the mean and *, **, ***, indicates significance at p < 0.05, p < 0.01 and p < 0.001, respectively.

## Abbreviations

SDF-1: Stromal derived factor-1, BrdU: 5 bromodeoxyuridine, E17: embryonic day 17, VZ: ventricular zone, ELISA: enzyme linked immunosorbent assay, DM: defined medium, LY: Lucifer yellow, Cx 43: connexin 43, GABA: gamma aminobutyric acid, Shh: sonic hedgehog, qRT-PCR: quantitative real time-polymerase chain reaction. LDH: lactate dehydrogenase, MTT: 3-(4,5-dimethylthiazol-2-yl)-2,5-diphenyltetrazolium bromide.

## Authors' contributions

JP: cell cultures, immunocytochemistry, imaging, qRT-PCR, ELISA, and quantitative analysis. CW: genotyping of mutants, generation of SDF-1 condition medium, qRT-PCR, axon tracings and overall assistance. LZ; microarray design and analysis. BN: project design, management, micro array analysis and preparation of the manuscript. All authors have read and approved the final version of the manuscript.

## Supplementary Material

Additional File 1 Characterization of E15 wild type and CXCR4-/- brain sections. Labeling of wild type (a-c) and mutant cortex (a'-c') for Ki67 (a, a'), MAP2 (b, b') and GABA (c, c') immunoreactivity; arrows in c, c' indicate GABA immunostaining in the MZ. (d) Line-scan analysis of mutant and wild type sections stained for Ki67. Because the pattern of staining was relatively homogenous across the cortical anlage, a line-scan analysis was performed to measure pixel intensity (Metamorph imaging software). Briefly, images were first processed for background subtraction. The average pixel intensity was then measured through a 50 μm radial column selected through the cortical axis. (e) Line-scan analysis of sections stained for GABA immunoreactivity; note the reduced pixel intensity in the MZ of mutant sections. Scale bar: 100 μm. PZ – proliferative zone, IZ – intermediate zone, CP – cortical plate, MZ – marginal zone, LV – lateral ventricle. Click here for file
