# Supplementary figures and images for: Stromal derived factor-1 exerts differential regulation on distinct cortical cell populations in vitro
Source: BMC Dev Biol. 2007 Apr 10;7:31. doi: 10.1186/1471-213X-7-31 (PMC1854892; doi:10.1186/1471-213X-7-31)

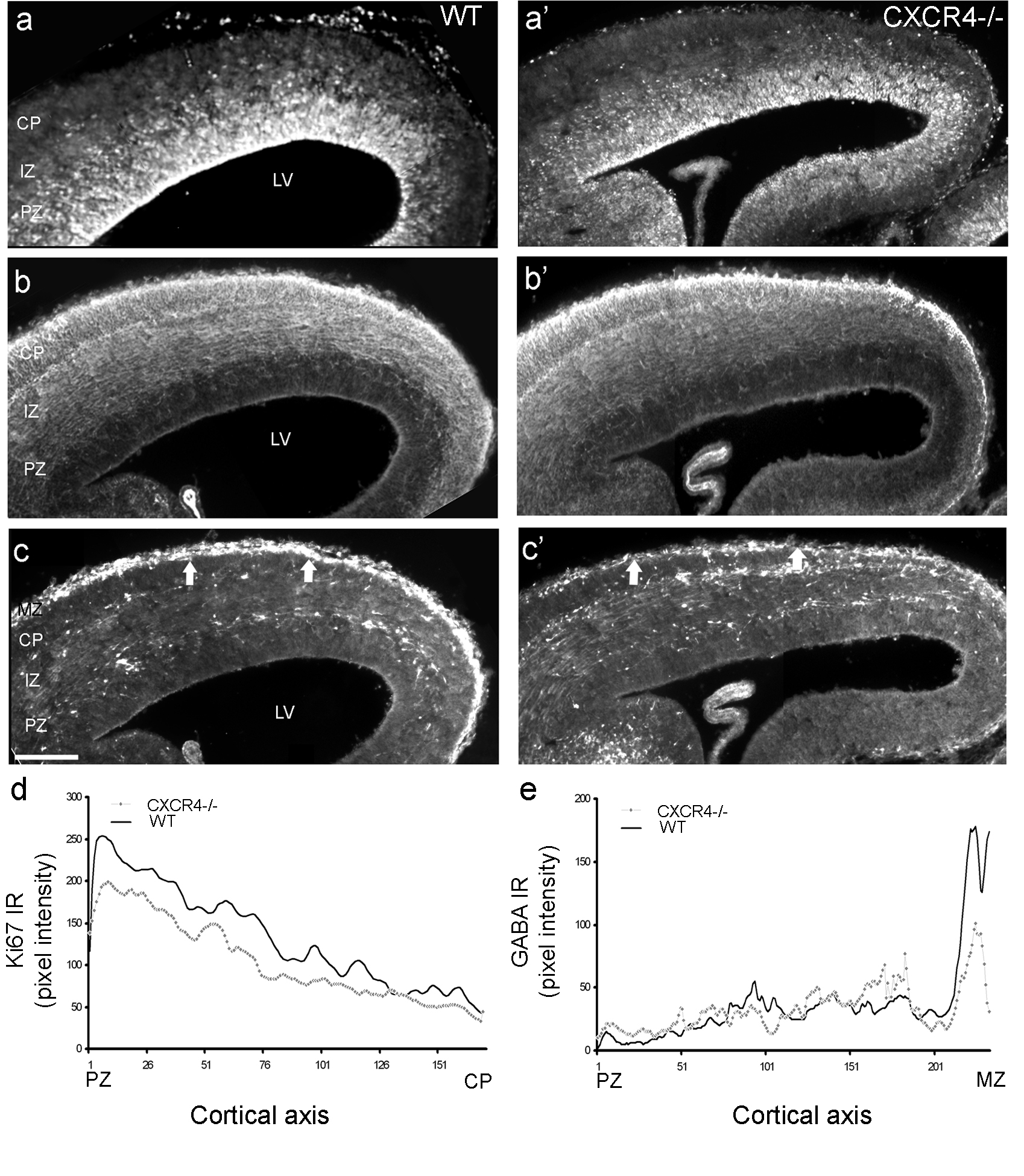

Supplement: Additional File 1 — Characterization of E15 wild type and CXCR4-/- brain sections. Labeling of wild type (a-c) and mutant cortex (a'-c') for Ki67 (a, a'), MAP2 (b, b') and GABA (c, c') immunoreactivity; arrows in c, c' indicate GABA immunostaining in the MZ. (d) Line-scan analysis of mutant and wild type sections stained for Ki67. Because the pattern of staining was relatively homogenous across the cortical anlage, a line-scan analysis was performed to measure pixel intensity (Metamorph imaging software). Briefly, images were first processed for background subtraction. The average pixel intensity was then measured through a 50 μm radial column selected through the cortical axis. (e) Line-scan analysis of sections stained for GABA immunoreactivity; note the reduced pixel intensity in the MZ of mutant sections. Scale bar: 100 μm. PZ – proliferative zone, IZ – intermediate zone, CP – cortical plate, MZ – marginal zone, LV – lateral ventricle. [file 1471-213X-7-31-S1.jpeg]
